# Federated Learning via Augmented Knowledge Distillation for Heterogenous Deep Human Activity Recognition Systems

**DOI:** 10.3390/s23010006

**Published:** 2022-12-20

**Authors:** Gad Gad, Zubair Fadlullah

**Affiliations:** 1 Department of Computer Science, Lakehead University, Thunder Bay, ON P7B 5E1, Canada; 2 Thunder Bay Regional Health Research Institute (TBRHRI), Thunder Bay, ON P7B 7A5, Canada

**Keywords:** deep learning, federated learning, knowledge distillation, human activity recognition, Internet of Things, hyperparameter tuning

## Abstract

Deep learning-based Human Activity Recognition (HAR) systems received a lot of interest for health monitoring and activity tracking on wearable devices. The availability of large and representative datasets is often a requirement for training accurate deep learning models. To keep private data on users’ devices while utilizing them to train deep learning models on huge datasets, Federated Learning (FL) was introduced as an inherently private distributed training paradigm. However, standard FL (FedAvg) lacks the capability to train heterogeneous model architectures. In this paper, we propose Federated Learning via Augmented Knowledge Distillation (FedAKD) for distributed training of heterogeneous models. FedAKD is evaluated on two HAR datasets: A waist-mounted tabular HAR dataset and a wrist-mounted time-series HAR dataset. FedAKD is more flexible than standard federated learning (FedAvg) as it enables collaborative heterogeneous deep learning models with various learning capacities. In the considered FL experiments, the communication overhead under FedAKD is 200X less compared with FL methods that communicate models’ gradients/weights. Relative to other model-agnostic FL methods, results show that FedAKD boosts performance gains of clients by up to 20 percent. Furthermore, FedAKD is shown to be relatively more robust under statistical heterogeneous scenarios.

## 1. Introduction

Developed in the year 2000 at the IEEE International Solid-State Circuits Conference (ISSCC), smartwatches saw rapid and wide adoption over the past years. As of 2020, one in every five Americans uses a smartwatch or a fitness band [1]. Human Activity Recognition (HAR) [2,3,4] is an emerging technology that employs low-power sensors found on mobile and wearable devices to detect, track, and analyze activity patterns. In order to facilitate remote health monitoring in rural communities, the utilization of HAR can automate data collection using wearables and Internet of Things (IoT) devices. While localized, distributed computing on such resource-constrained devices in the HAR systems is challenging, it has the potential to revolutionize medical analytics.

### 1.1. Deep Learning-Based HAR Systems

Following the success of Deep learning models in many domains such as computer vision [5], smart health [6,7,8], Natural Language Processing (NLP) [9], etc., Deep Learning (DL) has been used in HAR systems as a feature extraction method to improve the classification accuracy of activities using fewer sensors [10,11,12,13]. Proposed DL-based methods include using ResNet and BiLSTM [14] to extract spatial features of multidimensional signals, and sensor fusion with ConvTransformer [15] to achieve high-performance activity classification. In addition to performance gains, DL-based methods require little domain knowledge as they are able to learn directly from raw signals and fuse multi-sensor modalities. On the other hand, traditional Machine Learning (ML) methods often require expert knowledge and feature engineering both of which are expensive, and unique for a given set of sensors. Furthermore, DL methods’ expressive power as universal approximators is superior to that of traditional ML methods [16,17]. Deploying HAR DL-based models on edge devices still faces challenges such as memory footprint and power consumption. Additionally, data scarcity is another main obstacle for Deep learning as the availability of large datasets is usually a prerequisite for training high-quality deep learning models.

### 1.2. Sensors Used in Sensor-Based HAR Systems

Sensors are at the core of HAR systems. The diversity and quality of the sensors of a HAR system largely determine the accuracy of that system. Two of the widely used sensors in smartwatches and fitness bands are the Inertial Measurement Unit (IMU) and the Photoplethysmography (PPG) sensors. IMU is an integrated package that usually consists of an accelerometer, gyroscope, and magnetometer. These sensors measure the linear acceleration, rotation rate, and earth’s magnetic fields, respectively. An IMU that has all three sensors is referred to as a nine-axis IMU. Sometimes, an IMU does not have a magnetometer in which case it is called a six-axis IMU. The frequency of IMU (sampling rate) is manually tuned depending on the application; IMU frequency, therefore, ranges from 10 to several hundred Hz. Chung et al. in [18] studied sensor positioning impact on HAR performance and compared different IMU sampling rates and shows that a low-frequency (10 Hz) IMU signal can be effective for recognizing activities such as eating and driving. Using a higher sampling rate yields data with higher resolution and precision, which leads to more accurate analysis at the cost of higher resource consumption. Photoplethysmography (PPG) is an optical sensor used to measure heart rate using an infrared (IR) light sensor to measure blood flow, which is caused by the activity of the heart. The authors in [19] used raw PPG waveforms for Atrial fibrillation. Wrist-worn PPG sensors were employed for HAR in [20]. An accelerometer and heart rate derived from PPG were utilized to recognize activity during physical exercise in [21]. In this work, we collect gyroscope and PPG-based heart rate data to train the deep learning HAR system.

### 1.3. Federated Learning

Federated Learning (FL) [22,23] is a privacy-preserving distributed learning framework that enables learning effective models across participating devices. Most FL methods rely on sharing gradient to collaboratively train clients [23,24]. Gradient sharing-based FL implies that all participating models have the same architecture. In many real-world scenarios, however, devices with independently designed models want to collaboratively learn a task [25,26]. Model heterogeneity arises in areas such as health care, supply chain, and AI services. For example, in the context of healthcare, a group of medical institutions collaborating on some task may want to craft their own models to meet distinct specifications. Other reasons for model heterogeneity include privacy and intellectual property concerns. Knowledge Distillation (KD) [27] provides a model-agnostic way to transmit learned knowledge from one model to another. KD transfers knowledge by communicating models’ scores on a shared dataset instead of gradients. For instance, FedMD [26] employs a proxy dataset for knowledge distillation between heterogeneous models. Qinbin et al. [28] use a data-free KD approach by which clients send their models, trained on local data, to the server, and the server distills knowledge from clients’ models to a server-based generator. The generator is then broadcasted back to transfer its global view of the data distribution to local models.

**Figure 1:**
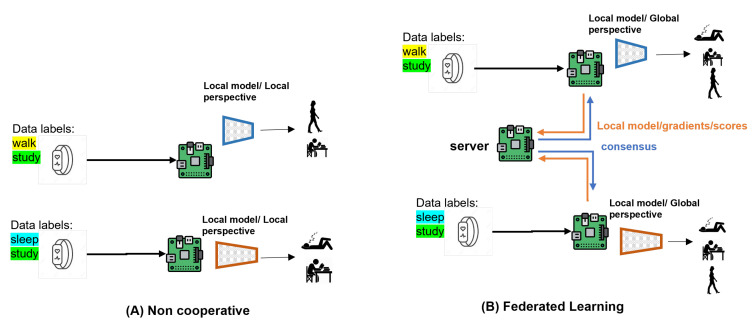
Non-cooperative local training produces local models with limited performance due to limited local training data. Federated Learning gives participating clients a holistic view of the combined data without explicitly sharing it, allowing a model to predict labels unseen in its train data. We go a step further in this work, collaboratively training models with independently designed model architectures.

Figure 1 highlights the advantages models obtain with FL vs. non-cooperative (central) training. A model trained on local data only has a local perspective, while a model trained with federated learning learns a holistic view of the data, enabling it to even successfully classify activities not found in its local data as we will show in our experiments.

### 1.4. Contribution

We propose a heterogeneous federated learning algorithm and evaluate it on self-collected wrist-mounted HAR and waist-mounted HAR datasets. Using augmented knowledge distillation, we show that our algorithm provides better convergence and accuracy in both experiments compared with other KD-based FL algorithms. Additionally, unlike standard FL (FedAvg), our proposed system FedAKD enables the participating clients to choose their own NN architecture and training configurations. Specifically,

1. The primary contribution of this work is FedAKD. A Federated Learning with Augmented Knowledge Distillation algorithm that enables collaborative training of clients with independently designed models;
2. FedAKD is evaluated on two sensor-based HAR datasets: a self-collected dataset (HARB) and a publicly available dataset (HARS) [29] to show the superior performance of FedAKD under different statistical conditions.

Our experiments show that FedAKD performs better than other heterogeneous FL algorithms, is more robust under extreme statistical heterogeneity scenarios, and has significantly less communication cost than gradient-based FL methods.

## 2. Related Work

### 2.1. HAR Systems and Sensor Fusion

Traditional Human Activity Recognition (HAR) systems have been extensively studied in recent work. Ref. [30] proposed a lightweight HAR system utilizing a Support Vector Machine (SVM) with fixed-point arithmetic for reducing the computational cost. Another SVM-based HAR system deployed on smartphones utilizing smartphone-based sensors was presented by [31]. The authors tested fitting a model on varying positions of smartphones. Ref. [32] developed a smartphone application that uses K-Nearest Neighbor (KNN) and the smartphone-based accelerometer sensor to detect activities from phone movement. Machine Learning (ML) based HAR methods learn shallow features from data leading to low HAR performance. HAR systems also use sensor fusion to integrate raw sensor measurements into more accurate measurements [33]. Sensor fusion is important in many scenarios to address problems such as limited spatial coverage and imprecision [34]. For instance, IMU integrates and fuses the readings of the accelerometer and the magnetometer to obtain the accurate orientation of an IMU [35]. In terms of the data processing level, sensor fusion can be divided into three types: data-level fusion, feature-level fusion, and decision-level fusion. In data-level fusion, data from multiple sensors are integrated. For example, the self-collected HARB dataset used in this work integrates heart rate pulses from the PPG sensor with gyroscope sensor readings in one input vector to the HAR system. In feature-level fusion, new features are calculated from the original features to provide a different perspective of the state being measured. For instance, to prevent misalignment issues [36], the magnitude of the accelerometer is usually calculated. Utilizing the acceleration forces measured in the three axes: $A_{x}$, $A_{y}$, and $A_{z}$, the orientation invariant acceleration magnitude $A_{m}$ is calculated as:

(1) $$A_{m}=\sqrt{A_{x}^{2}+A_{y}^{2}+A_{z}^{2}}$$

Decision-level fusion combines the decision of multiple classifiers into a common decision. As an example of decision-level fusion using ensemble learning, recent work [37] used an ensemble of deep learning models, where the predictions of many machine learning models are combined into a more accurate prediction, for elderly health monitoring based on smartphone sensors.

### 2.2. Deep Learning-Based HAR Systems

**Figure 2:**
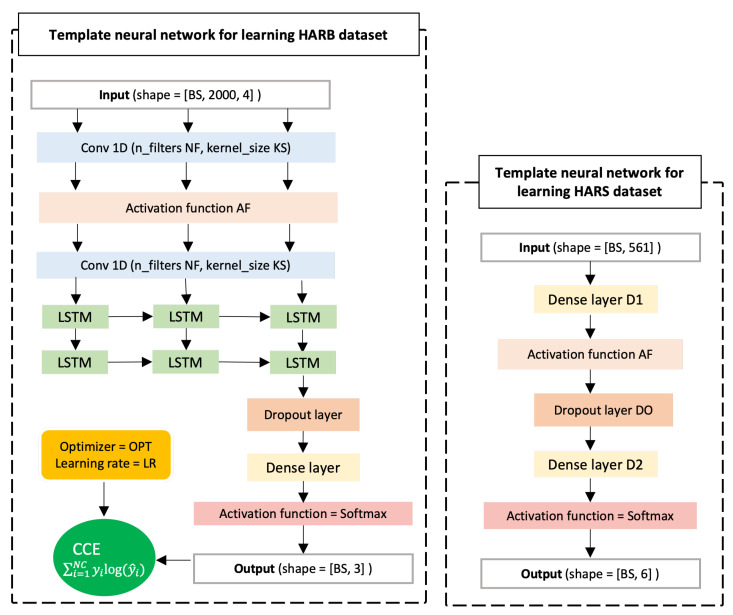
(**Left**) A template deep learning model used to derive heterogeneous models with various learning capacities for learning the HARB dataset in both centralized and federated learning settings; (**Right**) A template deep learning model is used to derive variant models to train on the HARS dataset in centralized and federated settings.

Deep Learning (DL) has seen significant growth in HAR due to its superior expressive power [17]. A Convolutional Neural Network (CNN)-based model presented by [21] used an accelerometer and Photoplethysmographic sensors for recognition of physical activity. Ref. [38] uses temporal and sensor attention combined with Long Short-Term Memory Units (LSTMs). Temporal attention focuses on important parts of the time series data while sensor attention focuses on important sensor modalities. The authors in [39] propose a computer vision approach for a Diver Activity Recognition (DAR) system on edge devices using a camera fixed in front of the driver. Reinforcement Learning (RL) approaches were also used in HAR. Ref. [40] proposed an online training RL-based policy gradient HAR system utilizing textile-based stretch and accelerometer sensors. In this work, we develop train deep learning models on sensor-based datasets based on the custom deep architectures shown in Figure 2 that consist of one-dimensional, CNN layers, LSTM units, dropout layer, and activation functions.

Additionally, wearable devices are expected to be comfortable to wear and easy to interact with. Therefore, HAR systems that incorporate three or more sensors [41,42] or that require the user to carry heavy recording equipment are not practical [43], and hence not suitable for HAR applications. Instead, Centinela [44] employs a chest-mounted strap with a single sensing device and a mobile phone to detect five activities: walking, running, sitting, ascending, and descending.

Compared to traditional HAR systems, DL-based HAR systems offer more powerful feature extraction capabilities, especially for extracting nonlinear relations [17]. This is attributed to various factors including backpropagation [45], a mechanism to reduce the mismatch between a model’s output and the desired output guided by a differential loss function, and activation functions [46,47], which introduce nonlinear transformations to the input data. Some activation functions use learnable parameters [47], which gives them the ability to assume different nonlinear functions, thus adapting better to various applications. However, the powerful feature extraction capability of DL comes at the cost of more power and memory consumption. To train high-quality DL models, large labeled datasets are needed to train on. Centralized training requires that users’ data are sent to a central server. Federated learning provides a private alternative to centralized training as it decouples the ability to do ML from the need to store data on the cloud. In the next section, we discuss some of the previous work carried out in the intersection between FL and HAR systems.

### 2.3. Federated Learning Applied to HAR Systems

Federated learning (FL) jointly trains clients utilizing a global model to update clients’ local models after a few local training iterations.

Sozinov et al. [48] study the impact of local data with corrupted labels on the performance of federated learning on synthetic and real-world datasets. The proposed FL algorithm can detect and reject erroneous clients. Additionally, they investigate the trade-off between communication cost and the complexity of models. The authors in [49] propose an FL algorithm that dynamically aggregates models’ weights according to the statistical distribution for each client by merging similar clients’ models in a layer-wise manner.

Many federated learning algorithms have been proposed to train distributed HAR systems instead of sending raw data to be analyzed at a server. Xiao et al. [50] designed an FL method where each client has a perceptive extraction network (PEN) which is composed of a feature network base on CNN blocks for feature extraction and a relation network based on LSTM and attention to mine global patterns in data. ClusterFL [24] is a similarity-aware FL system for HAR which exploits intrinsic similarities among users’ data. ClusterFL uses an alternating optimization approach to optimize model weights $w_{i}$, and a cluster indicator matrix *F* that quantifies the relationship between nodes. The loss function used by ClusterFL is given by

(2) $$\min_{W,F}\sum_{i=1}^{M}\frac{1}{N_{i}}\sum_{r=1}^{N_{i}}l\left(w_{i}^{T}x_{i}^{r},y_{i}^{r}\right)+\tau \mathrm{tr}\left(WW^{T}\right)-\gamma \mathrm{tr}\left(F^{T}WW^{T}F\right)$$

where *M*, $N_{i}T$, and $w_{i}$ represent the number of nodes, the local datasets size of *i*-th node, and the local weights of *i*-th node, respectively. The first term is the sum of empirical losses across nodes. The second and the third terms consist of the L2-norm and the K-means clustering. In the previous formulation, $F\in R^{M\times K}$ presents an orthogonal cluster indicator matrix, where if node k belongs to the q-th cluster, $F_{k,q}=\frac{1}{\sqrt{N_{q}}}$ and $F_{k,q}=0$, otherwise, where $N_{q}$ is the number of nodes in cluster *q*. τ and γ are hyperparameters, τ≥0 and γ>0.

Most FL paradigms use gradient averaging which does not account for participating clients that have heterogeneous model architectures, a real-world scenario that arises due to privacy concerns. Additionally, in the Internet of Things (IoT) domain, devices have limited computational and storage resources leading to deploying different models on edge devices according to their resources. Sending architecture-dependent data assumes all participating models have the same architecture. Knowledge Distillation (KD) [27] is a technique by which knowledge can be transferred from a trained model to a to-be-trained model. KD-based FL [26] presents a model-agnostic alternative to collaboratively train heterogeneous model architectures. Each client sends its scores on a shared dataset. The server calculates the consensus by averaging the received scores and broadcasts the consensus scores to clients. Clients train their models on a shared dataset employing consensus scores as labels.

## 3. Methodology

### 3.1. Human Activity Recognition with Fitness Band (HARB) Dataset

The first dataset utilized for evaluating the proposed system FedAKD is a self-collected sensor-based time-series HAR dataset that maps Gyroscope and Photoplethysmography (PPG) sensors to three activities: Walk, Study, and Sleep. We refer to this dataset as HARB for the rest of the paper. Data collection was carried out using the Mi band 4 fitness band, a commercial wearable device. We extracted Gyroscope readings and heart rate pulses calculated by the band based on the PPG sensor. In our experiment, we connect the Mi band 4 fitness band to a Raspberry Pi (RPI) computing board via Bluetooth Low Energy (BLE). Volunteers were invited to participate in data collection where they wear the band and power on the RPI and then start performing the target activity for a period of time. This period is called a data collection session, which ranges between 20–400 min, adjusted by the volunteer.

**Figure 3:**
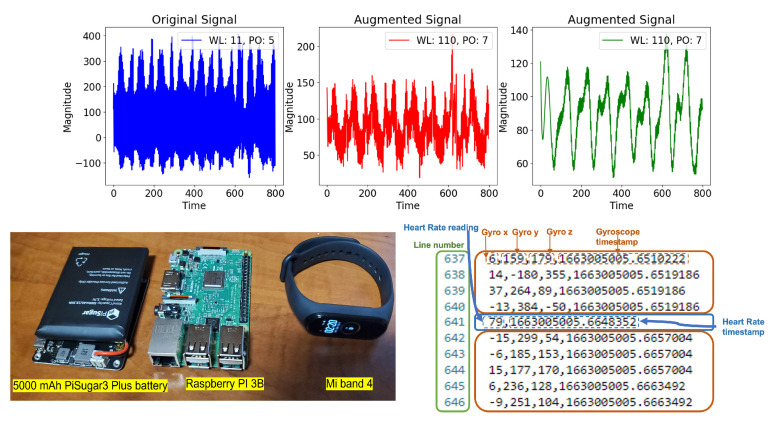
(**Top**) From left to right: Raw signal from the self-collected HARB dataset. An augmented version of the raw signal using a Sav–Gol filter using a window length of 11 and a polynomial order of 5. An augmented version of the raw signal using a Sav–Gol filter with a window length of 110 and a polynomial order of 7. The goal of augmentation is to balance the dataset. (**Bottom**) Right: Human Activity Recognition with fitness Band (HARB) dataset sample file format; (**Bottom**) Left Data collection equipment.

Figure 3 shows the data collection equipment. We used a battery-powered Raspberry Pi 3B. Volunteers put the RPI in their pockets while wearing the band during data collection to perform the target activity easily. The volunteer starts the data collection session by powering on the battery-connected RPI while wearing the Mi band 4. Once the RPI boots, a Linux on-boot service is triggered to start a Python script that uses the predefined MAC address and authentication key of the Mi band 4 to extract IMU and heart rate measurements. IMU has a frequency (i.e., sampling rate) of about 10–15 Hz, while the heart rate has a lower frequency of 2–3 Hz. This is because PPG raw data are processed by the wearable device before the calculated heart rate is produced. For each data collection session, a new file is automatically created in a data folder designated for that volunteer, and data are appended to it line by line. Because the frequency is inconsistent between the two data types, each line can either contain IMU measurements or heart rate values, in addition to the timestamp. Figure 3 (bottom) shows a screenshot of the contents of a sample dataset file. The data parser checks the number of comma-separated values in a given line. If two values are found, this line is considered to contain the heart rate value and the timestamp, respectively. If a line contains four values, they are considered to be the Gyroscope measurements on the *x*-, *y*-, and *z*-axes, and the timestamp of this measurement, respectively. Next, we discuss two challenges encountered during data parsing and training preprocessing, and how we address them:

- Frequency inconsistency: The frequency of the heart rate sensor is much less than that of the Gyroscope sensor. This is due to the fact that the retrieved heart rate readings are not raw sensor data but PPG signals processed by the fitness band itself. To address the issue of frequency inconsistency between both data streams, heart rate readings were interpolated to increase their frequency to match that of the Gyroscope sensor;
- Noisy heart rate measurements: Both Gyroscope and heart rate measurements are integers, whereas timestamp is a float value. Since the Gyroscope measures angular velocity, it can take negative values where the sign indicates the direction of rotation. On the other hand, heart rate should not take negative values. We found that the fitness band sometimes returns negative heart rate readings. Such values are considered to be noise and completely ignored by the parser.

### 3.2. Human Activity Recognition with Smartphone (HARS) Dataset

The second dataset which we used to evaluate the proposed FL system FedAKD is a smartphone-based HAR dataset [29] that maps internal sensors to six activities: Walk, Walk up-stairs, Walk down-stairs, Stand, Sit, and Lay. We refer to this dataset as HARS for the rest of the paper. Although inertial sensors produce a time-series signal, HARS is a tabular dataset where each record is a 561-feature vector calculated using time and frequency domain variables. First, noise filters were applied to the raw signal. The authors then sampled the signal using fixed-width sliding windows that have a width of 128 readings and an offset of 64 readings. Time and frequency domain variables were calculated from which a 651-feature vector is constructed [29].

Table 1 shows details about each dataset, while HARS employs 50 Hz sensors, and HARB relies on a device that uses low-frequency sensors (2–15 Hz). Other differences are the modality of the data and the employed sensors. HARB is a time-series dataset while HARS is a tabular dataset. Regarding the used sensors, HARS uses smartphone-embedded inertial sensors while HARB utilizes the Gyroscope and Photoplethysmography (PPG) sensors.

### 3.3. Data Preprocessing and Local Datasets Distribution

**Figure 4:**
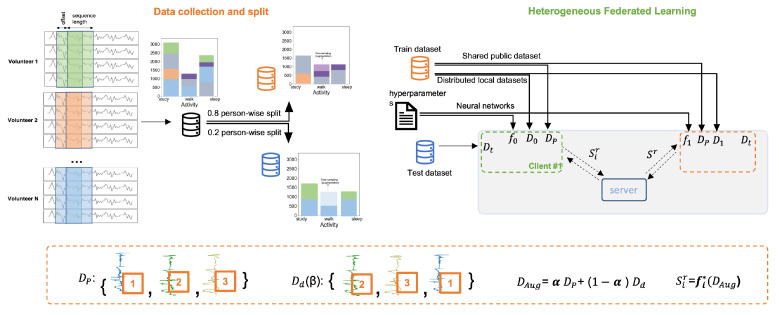
An overview of data preprocessing and splitting of the self-collected HARB dataset, and how each component is being utilized in the proposed heterogeneous Federated Learning system. The dotted box in the bottom explains the augmentation mechanism used in this work which is based on mixup augmentation and permutation. Signal colors and the associated number represent the index of each sample.

**Figure 5:**
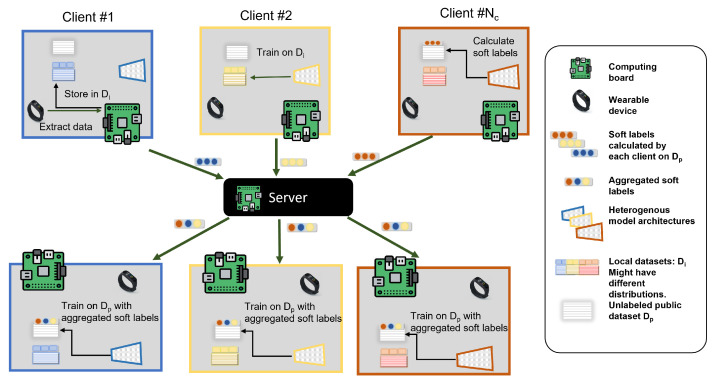
An overview of the proposed heterogeneous federated learning with knowledge distillation architecture. Each client owns a local dataset, an independently designed model, and a shared dataset. By utilizing knowledge distillation, clients use the shared dataset to transfer the knowledge they learned from local datasets by communicating their soft labels on the shared dataset with all clients. We propose an additional step where the shared dataset $D_{p}$ is augmented to be $D_{Aug}^{r}$ in order to enhance performance.

Figure 4 provides an overview of the FL experiment on the HARB dataset starting from sampling the raw time-series data of each volunteer, labeling data, and splitting data subject-wise into train and test sets. Including all subjects in both train and test sets results in an information leak which causes test accuracy to be high; however, the model performs much worse on unseen data from new subjects, which is illustrated in [51]. The sizes of the train and test sets of both datasets are shown in Table 2. We applied augmentation to the walk data of the HARB dataset using the Sav–Gol filter [52] to double its sizes to balance the dataset before applying centralized training. The 10 models designed for the HARB FL experiment were first trained on the dataset for 20 epochs and a batch size of 32 to report its centralized training. Likewise, the deep models designed for the FL experiment on the HARS dataset were trained with the same number of epochs and batch size in a centralized manner. Since both datasets are balanced, accuracy is used as a performance measure for both centralized training and FL training. If the dataset is unbalanced, metrics such as macro-F1 score and balanced accuracy can be used to reflect the performance of the model on minority classes [53]. Figure 5 gives a closer look into the proposed heterogeneous federated learning algorithm.

**Figure 6:**
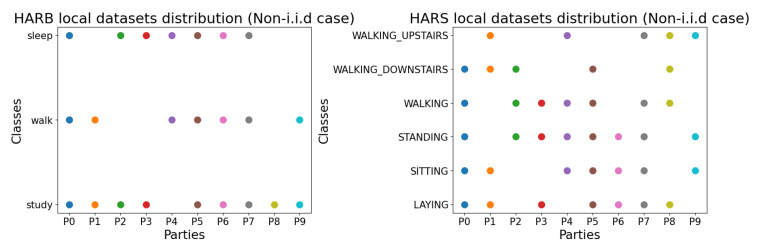
From left to right, the figures show the non-i.i.d distribution in the federated learning experiments of HARB and HARS datasets, respectively. Points show whether a party/client possesses a particular class in his local dataset $D_{i}$ or not. For example, in the HARS dataset, the party P2 has three classes out of six. All clients are tested against all classes (The test dataset $D_{t}$ is the same for all parties). Each color in the scatter plot refers to a client.

The centralized training accuracy per model for the HARB and HARS datasets is reported along with models’ hyperparameters in Table 3 and Table 4, respectively. After that, the FL experiment commences in which each of the 10 clients uses only 20 samples per class as his local dataset; however, the whole test set in Table 2 is used as the test set to calculate the model’s test accuracy during the FL experiment. Figure 6 shows the non-i.i.d distribution of HARB and HARS datasets. In the non-i.i.d, local datasets drop some classes. For example, the local dataset of the second client/party (P1) has only four classes, which mean a total of 80 samples in his local dataset (assuming 20 samples per class).

To distill the knowledge between clients, we need a shared dataset that has a distribution similar to the local datasets’ distributions that the clients are trying to collaboratively learn. In both datasets, 100 sample points from the train set were employed as a public dataset where they are shared between clients and used to calculate soft labels for knowledge distillation. In a real-world scenario, the public dataset is shared and broadcasted at the beginning of the FL processing by the server (e.g., fitness band company) as a medium for communication.

### 3.4. Model Selection

The need to apply Federated Learning (FL) on heterogeneous model architectures arises for various reasons. For instance, a smartwatch manufacturer who releases a new device annually which has more resources and thus can run more expensive deep Neural Networks (NN). This manufacturer may want to apply FL among these devices to make use of the data that is generated on users’ devices while protecting users’ privacy. We test our FL system, FedAKD, on a group of heterogeneous NNs with a number of parameters ranging between 1.9k to 30k for the HARB experiment, and ranging between 4k to 291k parameters for the HARS dataset. There are different ways to build the ten heterogeneous models. We first designed a custom template deep learning model for each dataset. The design considered the input and output sizes, utilizing different sequence processing units such as Long Short Term Memory (LSTM) units [54] and one-dimensional, Convolutional Neural Network (1DCNN) layers [55], and using different types of activation functions including Relu, Sigmoid, and Tanh to extract nonlinear feature relations. To prevent overfitting [56], we use a dropout layer [57] whose drop rate is also tunable. A distinct feature of FedAKD is that it allows clients also to choose the optimizer, which is usually controlled by the server in gradients/weights-based FL methods. We set the optimizer in the model variants to be built to be one of the following three optimizers: Stochastic Gradient Descent (SGD) [58], Adam [59], and RMSprop [60].

The models participating in the HARB experiment were selected using the template model shown in Figure 2 to the left. One hundred variant models of that template were generated and we use hyperparameter tuning to sample ten models that cover the range of performance and learning capacity of the whole group as judged by the relative test accuracy and the number of parameters. That is, we sort models based on their test accuracy and uniformly sample ten models where the first is the highest performing and the last is the least performing in the whole group. A correlation can be observed between the number of parameters and models’ performance as shown in Table 3. Other factors that impact the performance of the model in addition to the number of parameters and the model architecture include the used learning rate and the optimizer.

In model selection for the HARS experiment, we first build a model with random hyperparameters based on the template model shown in Figure 2 to the right, and manually tune its hyperparameters until we obtain good performance (>90%) on the HARS dataset. The other models were derived from the initial model by randomly increasing/decreasing numerical values such as the number of units in dense layers and the drop rate, and randomly selecting other categorical hyperparameters such as the optimizer and the activation functions. Table 4 shows the architecture details of the models and their sizes.

The goal of the model selection step is to obtain 10 models that have distinct learning capacities to evaluate FedAKD’s ability to collaboratively boost the performance of these models. Instead of tuning model hyperparameters to find a good balance between size and performance, another approach called feature selection trains a particular model architecture is trained on different feature sets. This method is part of the feature engineering phase which is usually used in ML-based models. However, DL-based models are able to implicitly select the important features based on how much each feature contributes to the output. Feature selection is also used in centralized training of DL-models [61]; however, this is out of the scope of this work as we are interested in evaluating FedAKD in the FL setting.

### 3.5. Federated Learning

First, we present the system annotation of federated learning and then describe the proposed Federated Learning with Augmented Knowledge Distillation (fedAKD).

#### 3.5.1. Problem Definition

Consider $C=C_{1},C_{2},\ldots ,C_{N_{c}}$ to be the set of clients in a federated learning task, where $N_{c}$ is the number of clients, and $C_{i}$ is the $i^{th}$ client. $C_{i}$ has a local private dataset $D_{i}$, a shared public dataset $D_{p}$ to transfer knowledge, and an independently designed model $f_{i}$. $D_{i}$ is a supervised dataset in which the $j^{th}$ sample $D_{i}^{\left[j\right]}=\left\{X^{\left[j\right]},Y^{\left[j\right]}\right\}$. $D_{p}$ is an unsupervised dataset in which the $j^{th}$ sample $D_{p}^{\left[j\right]}=\left\{X_{p}^{\left[j\right]}\right\}$. The task is to train $f_{i}$ on $D_{i}$ without explicitly sharing data to reach the would-be performance if $f_{i}$ was trained on the collected private dataset $D=\{{D_{i}}_{i=1}^{N_{c}}\}$.

#### 3.5.2. Federated Learning with Augmented Knowledge Distillation

**Figure 7:**
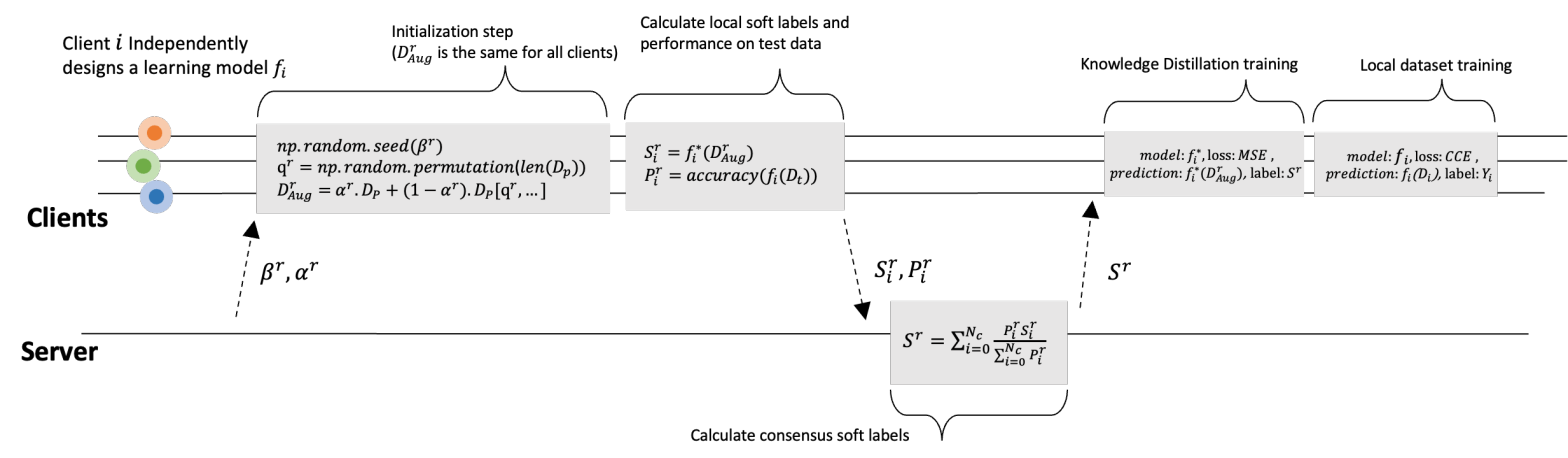
FedAKD timeline at communication round *r*. $f_{i}$ is the independently designed model of client *i*. We remove the last layer (Softmax) from $f_{i}$ to obtain $f_{i}^{*}$ which produces the soft label $S_{i}^{r}$.

FedAKD algorithm consists of eight steps that can be summarized as:

**1.**  **The server broadcasts**$\beta^{r}$**and**$\alpha^{r}$**:**$\beta^{r}$ is an integer used to seed the permutation algorithm to generate the same permuted version of $D_{p}$, called $D_{d}$, across clients. Then, $\alpha^{r}$ is used to apply mixup augmentation [62]
**2.**  **Clients calculate**$D_{Aug}^{r}$**from**$D_{p}$**:** Client i uses $\beta^{r}$ to seed the permutation algorithm. For example, Figure 7 shows how this is carried out in Numpy ( Python’s numerical library). Then, $\alpha^{r}$ is used to generate $D_{Aug}^{r}$ as follows:
  (3) $$D_{Aug}^{r}=\alpha^{r}D_{d}^{r}+\left(1-\alpha^{r}\right)D_{p}$$
**3.**  **Clients calculate**$S_{i}^{r}$**and**$P_{i}^{r}$**:** Client i calculates the soft labels $S_{i}^{r}$ as follows:
  (4) $$S_{i}^{r}=f_{i}^{*}\left(D_{Aug}^{r}\right)$$
  where $f_{i}^{*}$ is the same as $f_{i}$ with the Softmax layer removed. FedAKD clients employ $S_{i}^{r}$ to send their local knowledge to the server. Then, the client also calculates the performance $P_{i}^{r}$ of $f_{i}$ on $D_{t}$ to weigh clients’ contributions proportional to their performance.
**4.**  **Clients send**$S_{i}^{r}$**and**$P_{i}^{r}$**to the server:** Each client i sends its local soft labels $S_{i}^{r}$ and its performance $P_{i}^{r}$ calculated on the test dataset $D_{t}$ to the server.
**5.**  **The server aggregates all soft labels into**$S^{r}$**:** The server calculates the consensus soft labels $S^{r}$ from clients’ soft labels $S_{i}^{r}$ weighted by $P_{i}^{r}$ as follows:
  (5) $$S^{r}=\sum_{i=1}^{N_{c}}\frac{P_{i}S_{i}^{r}}{\sum_{k=0}^{N_{c}}P_{k}}$$
**6.**  **The server broadcasts**$S^{r}$**:** The server broadcasts the consensus soft labels $S^{r}$ to all clients to use them as labels for the augmented dataset of round *r*.
**7.**  **Knowledge distillation training:** Clients use the received global/consensus soft labels $S^{r}$ as the ground truth labels of the generated augmented dataset $D_{Aug}^{r}$ using Mean Squared Error (MSE) loss for local epochs E1. The goal of this step is to train clients (also called students) to produce soft labels $S_{i}^{r}$ that match the server’s (also called the teacher) soft labels $S^{r}$.
**8.**  **Local dataset training:** Clients train on their labeled datasets $D_{i}$ using Categorical Cross Entropy (CCE) loss for local epochs E2.

**Figure 8:**
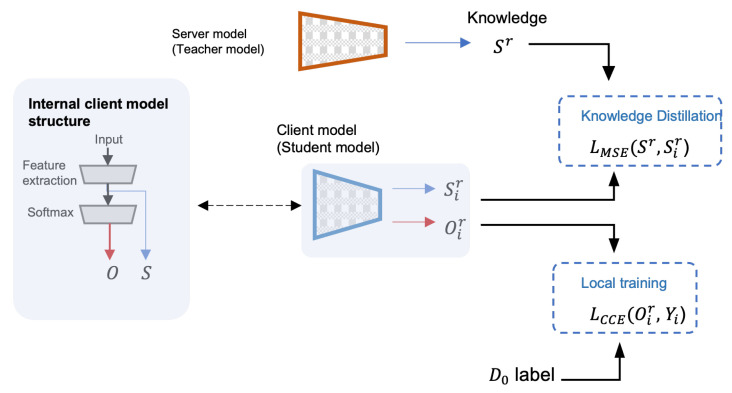
The Knowledge Distillation mechanism. The soft labels of a trained teacher model are used to distill knowledge to a student model.

Next, we discuss the proposed algorithm further in light of the two Figure 7 and Figure 8. In Figure 8, the last two steps are illustrated. The student model produces two outputs: $O_{i}^{r}$, and $S_{i}^{r}$. $O_{i}^{r}$ is a probability distribution since it is the output of a Softmax layer (The elements of the vector $O_{i}^{r}$ sum up to 1). $O_{i}^{r}$ and $S_{i}^{r}$ are referred to as hard labels and soft labels, respectively. We apply MSE loss to the teacher’s soft labels (as labels) and the student’s soft labels ( as predictions) to update the weights of the student’s model. After that, CCE loss is used to train the student model on the local dataset $D_{i}$ using the student’s hard labels $O_{i}^{r}$ to update the same weights. In Figure 7, a timeline of FedAKD shows the operations performed by the server and the clients at global round *r*. The timeline starts from left to right. Note that each client independently designs his learning model $f_{i}$, which is not the case in gradient/weights-based FL methods such as FedAvg [23], where the server controls the architecture of the model $f_{i}$. The eight steps described earlier are shown as five gray blocks that represent the computation operations performed by the server or the clients, and three dashed arrows, representing communication operations.

Compared to prior work [26], our algorithm FedKD introduces two main modifications:

- Performance-weighted Averaging: Unlike [63], where the server weights clients’ gradients proportional to the size of their local datasets, and [26] which uses uniform averaging. The server in FedAKD weighs the soft labels of the i-th client proportional to its performance (accuracy) $P_{i}^{r}$ on the shared test dataset $D_{t}$ at global round *r*;
- Mixup+Permutation Augmentation: FedAKD utilizes a server-controlled permutation in addition to mixup augmentation [62] to introduce variance to the public dataset and distill more knowledge. Our experiments show that this technique improves performance, especially in the non-i.i.d scenario compared to not using augmentation [26].

## 4. Results and Discussion

**Figure 9:**
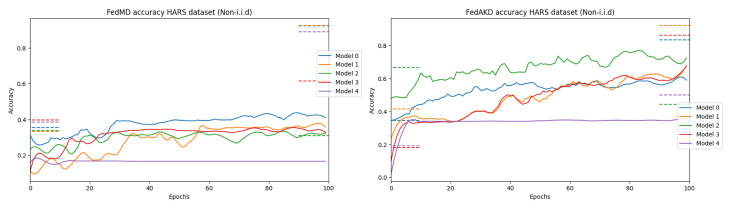
Ten heterogeneous models are trained in an FL setting using FedAKD (ours) and FedMD [26]. From left to right, the plots show five models (out of ten) trained collaboratively using FedAKD and FedMD, respectively, under the non-i.i.d case. It can be observed that models achieve better using our method. The five models shown here are the first five models in Table 4. The dashed line to the left and the right of each graph represent models’ performance on their local private dataset and models’ performance on all the local datasets combined, respectively. In all experiments, every client has 20 samples only per activity (as shown in Figure 6) as his $D_{i}$, and $|D_{p}|=100$. The lines are smoothed using the Sav–Gol filter to show the trend more clearly.

In this section, we evaluate the proposed FL algorithm FedAKD on two HAR datasets HARS and HARB. The template models employed for the HARS and HARB datasets are shown in Figure 2 to the right and left, respectively. Ten variant models with various sizes and hyperparameters are derived from these template models to evaluate FedAKD on both datasets using heterogeneous model architectures. First, models are trained in a centralized manner on their respective datasets to assess their learning capacity. The training data which were used in centralized training is distributed and 20 samples per class are given to each client as his local dataset (Note that the number of classes available to each client is different in the non-i.i.d case). Each model is trained in a centralized manner on its own local dataset and trained on the collected local datasets. Models’ performance under these two training settings forms the lower and upper bound for our FL experiment, respectively. That is, the goal of FL is to push the performance of each model beyond its local effort and towards the would-be-performance, if all local datasets were combined and made available to train on. These lower and upper bounds are shown as horizontal dash lines to the left and right, respectively, on the plots of Figure 9.

The model architectures, sizes, and the obtained centralized training performance (accuracy) are shown in the Table 3 and Table 4 for the HARB and HARS datasets, respectively. For the HARS dataset, it can be observed that the best-performing model achieved 95.4%, while the least-performing model achieved 34.5%. These two models have 17K and 242K parameters, respectively. This phenomenon is known as overparameterization, and it means that the model is so complex that it memorizes data and fails to generalize [64]. Choosing the optimal hyperparameters including the size of the used models is beyond the scope of this work as our focus is finding the accuracy gains achieved using FedAKD relative to other FL methods on both datasets using heterogeneous models. For the HARB dataset, Table 3 shows the accuracy of each model in centralized training. The best performing model achieved 67.8%, while the least performing model achieved 58.6%.

Table 5 summarizes the main numerical results of this work. It shows the average accuracy gains of our proposed FL system FedAKD and FedMD [26] on both datasets. The number of samples in the local datasets is set to 20 per class (non-i.i.d distribution is shown in Figure 6, and the size of the shared public dataset is set to 100. FedAKD achieves better average accuracy gains. For the HARS dataset, FedAKD obtained 25.4% and 27.5% under the i.i.d and non-i.i.d cases, respectively. FedMD achieved 24.5% and 7.2% under the i.i.d and non-i.i.d cases, respectively. FedAKD performs significantly better than FedMD under the non-i.i.d case achieving an extra 20.3%. Considering the HARB dataset, FedAKD obtained 12.7% and 0.4% under the i.i.d and non-i.i.d cases, respectively. On the other hand, FedMD obtained 11.5% and −2.7% under the i.i.d and non-i.i.d cases, respectively. The negative average accuracy gains obtained by FedMD are attributed to the complexity of the non-i.i.d case and the low predictive power of the HARB dataset which in turn is attributed to the fact that it is based on low-frequency sensors. Again, FedAKD outperforms FedAMD on the HARB dataset by achieving a positive average accuracy gain under the non-i.i.d case.

### 4.1. FedAKD Communication Cost

In terms of communication efficiency, FedAKD is much more efficient compared with FedAvg [23] and other gradients/weights-based FL methods. In the global communication round *r*, FedAKD clients and server communicate $2\left|D_{p}\right|*n_{classes}+\left|P_{i}^{r}\right|$. Since $\left|P_{i}^{r}\right|$ equals $\left|D_{p}\right|$, the communication cost of each round of FedAKD is given by $\left|D_{p}\right|\left(2n_{classes}+1\right)$. On the other hand, FedAvg sends the weights of clients’ local models to the server and broadcasts the weights of the global models back to clients. For instance, assume model 4 in Table 4 which includes the models for the HARS dataset is used by all clients in a FedAvg scenario. In each global round, each client will send 113k float values (the size of model 4) to the server and receive the aggregated model, which has the same size. Therefore, each client will suffer a communication overhead of 226k × 8 Bytes = 1.8 MB (using FedAvg), compared to 100 × (2 × 5 + 1) × 8 Bytes = 8.8 KB (using FedAKD).

### 4.2. FedAKD Performance Analysis

**Figure 10:**
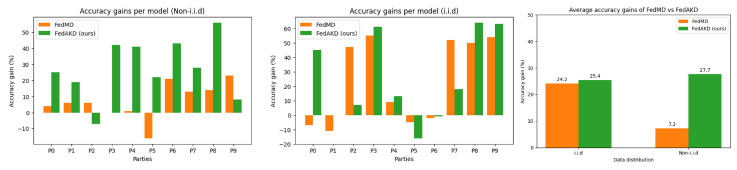
Comparison of individual models’ accuracy gains achieved by our proposed method: FedAKD and FedMD [26] under i.i.d (**middle**) and non-i.i.d (**left**) cases on the HARS dataset. The bar plot to the (**right**) shows the average accuracy gains (across models) under both statistical conditions. FedAKD performs significantly better than FedMD in the non-i.i.d case.

Our experiments show that both of our methods outperform FedMD [26] under non-i.i.d scenarios. Figure 9 shows the test accuracy performance of the first five heterogeneous models in Table 4 using FedMD [26] (to the left), and our proposed method FedKD (to the right). Additionally, the bar plots in Figure 10 show the accuracy gains of each of the ten models in the FL experiment on the HARS dataset. It can be seen that the performance of individual models under FedAKD is better than their performance under FedAKD, especially, in the non-i.i.d case (left).

## 5. Conclusions

In this paper, we propose FedAKD, a federated learning algorithm for collaborative training of heterogeneous deep learning models. FedAKD is based on knowledge distillation, a student–teacher paradigm to train a model using the knowledge that a trained model has. We evaluate FedAKD on two human activity recognition datasets: HARS, a tabular dataset extracted from smartphone-embedded inertial sensors, and HARB. a self-collected time-series dataset extracted from the Gyroscope and Photoplethysmography sensors of a fitness band. The considered FL experiments use heterogeneous deep learning models with sizes (number of parameters) ranging from 1.9k to 30k for the HARB dataset, and ranging between 4k to 291k 273 parameters for the HARS dataset. In addition to model heterogeneity, FedAKD is evaluated under extreme statistical heterogeneity in which some clients are tested on activities/labels whose corresponding samples are not found in their local datasets; therefore, the knowledge needed to classify these labels has to be distilled from the other clients.

Compared with FedAvg [23], our proposed FL algorithm is much cheaper in terms of communication cost. In the considered FL experiment on the HARS dataset, we show that FedAKD is 200X more communication-efficient than FedAvg; FedAKD devices communicate a total of 8.8 Kilo Bytes (KB) vs. 1.8 Mega Bytes (MB) on average if devices were to use FedAvg.

Compared with other knowledge distillation-based FL algorithms [26] which enable FL of heterogenous models, our proposed algorithm FedAKD achieves higher accuracy gains for most participating models and significantly higher average accuracy gain across models on both datasets under i.i.d and non-i.i.d conditions. Specifically, for the HARS dataset, FedAKD obtained 25.4 % and 27.5 % under the i.i.d and non-i.i.d cases, respectively, while FedMD achieved 24.5 % and 7.2 % under the same statistical scenarios. That is, FedAKD achieves an extra 20% of average accuracy gains compared with FedMD. This boost in performance is attributed to the fact that FedAKD uses augmentation to generate a new variant of the public dataset in each communication round, which helps distill knowledge more efficiently.

## 6. Limitations and Future Work

In our FL experiments, the public dataset $D_{p}$ was taken from the training set of the respective dataset. A better approach is to choose $D_{p}$ as a different dataset that has a similar distribution to the considered local dataset. For instance, in [26], the authors used MNIST as a public dataset to train heterogeneous models on the local dataset FEMINIST. In another experiment, when training models on CIFAR100, they employed CIFAR10 as the public dataset. In our approach, we assumed that the public dataset (which contains only 100 samples) is made available to clients by the server at the beginning of FedAKD. In a real-world scenario, a company will collect some data and store them on its devices to be used as a public dataset during FL. This way, the company can protect users’ data (by not using part of these data as a public dataset), and at the same time, the stored public dataset will have a distribution that is similar to the distribution of the local data that will be collected by users (since they are both collected using the same sensors).

In future work, we would like to integrate privacy-preserving techniques such as Differential Privacy (DP) with our Augmented Knowledge Distillation (AKD) algorithm. Additionally, more analysis could be conducted on class-level performance under this knowledge–distillation FL paradigm. Finally, we would like to evaluate FedAKD on other data modalities (like images) and applications (like medical analysis).

## Figures and Tables

**Table 1:** HARB and HARS datasets sizes.

Dataset	Human Activity Recognition Using Smartphone (HARS)	Human Activity Recognition Using Fitness Band (HARB)
Train set size	6616 samples	3000 samples
Test set size	2947 samples	2000 samples
Local dataset size (per client): i.i.d	20×6=120 samples	20×3=60 samples
Local dataset size (per client): non-i.i.d	20× number of classes (≤6)	20× number of classes (≤3)

**Table 2:** HARB and HARS datasets characteristics.

**Dataset**	Human Activity Recognition using Smartphone (HARS)	Human Activity Recognition using fitness Band (HARB)
**Availability**	Publicly available dataset ^1^	Self-collected. Samples are available online ^2^.
**Source**	Waist-mounted smartphone	Wrist-mounted fitness band
**Sensors**	Smartphone inertial sensors	Photoplethysmography (PPG) sensor and Gyroscope
**Sensors frequency**	50 Hz	Hear rate: 2 Hz, Gyroscope: 15 Hz
**Data modality**	Tabular (A 561-feature vector from the time and frequency domain variables)	Time-series (sampled in fixed-width sliding windows)
**Number of Activities**	3	6
**Activities/Classes**	Walk, Study, Sleep	Walk, Walk up-stairs, Walk down-stairs, Sit, Stand, Lay

^1^https://www.kaggle.com/datasets/uciml/human-activity-recognition-with-smartphones (accessed on 10 December 2022). ^2^ https://github.com/gadm21/FedAKD (accessed on 10 December 2022).

**Table 3:** Architecture details of the deep learning models participating in the HARB dataset FL experiment.

Model ID	NF	KS	NCL	NLL	AF	OPT	LR	Size (Number of Parameters)	Centralized Training (%)	Accuracy Gain (%)		Accuracy Gain (%)	
										i.i.d		Non-i.i.d	
										FedMD	FedAKD (Ours)	FedMD	FedAKD (Ours)
Model 0	20	5	3	2	Relu	Adam	1 $\times \;10^{-4}$	28,016	58.6	0	20	−6	−3
Model 1	20	5	1	1	Sigmoid	Adam	7 $\times \;10^{-5}$	7064	67.8	22	38	−8	−5
Model 2	20	9	2	1	Relu	Adam	4 $\times \;10^{-5}$	11,004	60	−11	13	−12	−9
Model 3	10	9	2	2	Relu	RMSprop	1 $\times \;10^{-5}$	23,556	60.9	−1	6	9	12
Model 4	20	9	2	2	Sigmoid	RMSprop	7 $\times \;10^{-5}$	5344	63.1	8	-7	2	6
Model 5	5	9	3	3	Tanh	Adam	1 $\times \;10^{-4}$	30,601	58.9	42	27	2	5
Model 6	20	9	3	1	Relu	RMSprop	1 $\times \;10^{-5}$	8744	68	18	20	−20	−17
Model 7	10	18	2	3	Sigmoid	Adam	1 $\times \;10^{-5}$	3544	59.9	14	2	−14	−11
Model 8	5	9	1	3	Sigmoid	SGD	4 $\times \;10^{-5}$	12,189	61.2	22	23	5	8
Model 9	20	9	1	3	Sigmoid	SGD	4 $\times \;10^{-5}$	1944	57.5	1	−15	15	18

**Table 4:** Architecture details of the deep learning models participating in the HARS dataset FL experiment.

Model ID	D1	AF1	DO	D2	OPT	LR	Size (Number of Parameters)	Centralized Accuracy (%)	Accuracy Gain per Model (%)			
									i.i.d		Non-i.i.d	
									FedMD	FedAKD (Ours)	FedMD	FedAKD (Ours)
Model 0	290	relu	0.1	340	Adam	1 $\times \;10^{-3}$	291k	85.1	−7	45	4	25
Model 1	240	elu	0.25	300	Adam	1 $\times \;10^{-4}$	242k	34.5	−11	0	6	19
Model 2	200	selu	0.15	270	Adam	1 $\times \;10^{-5}$	207k	72.4	47	7	6	−7
Model 3	93	relu	0.2	200	RMSprop	1 $\times \;10^{-5}$	131k	87.1	55	61	0	42
Model 4	99	tanh	0.1	170	RMSprop	1 $\times \;10^{-4}$	113k	94.4	9	13	1	41
Model 5	90	elu	0.15	120	Adam	1 $\times \;10^{-3}$	78k	94.9	−5	−16	−16	22
Model 6	20	relu	0.25	70	RMSprop	1 $\times \;10^{-3}$	40k	86.9	−2	−1	21	43
Model 7	7	selu	0.1	30	Adam	1 $\times \;10^{-4}$	17k	95.4	52	18	13	28
Model 8	5	tanh	0.15	10	SGD	1 $\times \;10^{-3}$	5.5k	39.1	50	64	14	56
Model 9	5	tanh	0.25	8	SGD	1 $\times \;10^{-5}$	4.5k	87.4	54	63	23	8

**Table 5:** Summary of the numerical results of the FL experiments on both the HARB and HARS datasets. Our proposed FL algorithm FedAKD outperforms FedMD on both datasets under i.i.d and non-i.i.d statistical scenarios.

Average Accuracy Gains of Federated Learning Experiments (%)					
Dataset		HARS		HARB	
Data distribution		i.i.d	Non-i.i.d	i.i.d	Non-i.i.d
Method	FedMD	24.5	7.2	11.5	−2.7
	lFedAKD (ours)	25.4	27.5	12.7	0.4

## Data Availability

The scripts containing all the analysis carried out in this work are available at https://github.com/gadm21/FedAKD (accessed on 10 December 2022). The HARS dataset and part of the HARB dataset can also be found in the mentioned repository.

## Abbreviations

The following abbreviations are used in this manuscript:

FL	Federated Learning
HAR	Human Activity Recognition
i.i.d	Independent and identically distributed
KD	Knowledge Distillation
*E*	The number of local epochs
*R*	The number of global communication rounds
$\beta^{r}$	An integer used to seed the permutation of $D_{p}$ at round *r*
$\alpha^{r}$	constant ∈[0,1] used to calculate $D_{Aug}^{r}$
C	a set of clients/devices participating in FL
$C_{i}$	The $i^{th}$ client
$f_{i}$	Independently designed deep model of the $i^{th}$ client
$f_{i}^{*}$	$f_{i}$ minus the last softmax activation layer
$D_{i}$	The $i^{th}$ local dataset of the $i^{th}$ client
$\left\{X^{\left[j\right]},Y^{\left[j\right]}\right\}$	The $j^{th}$ sample pair in a local dataset
$D_{t}$	test dataset (shared)
$D_{p}$	the public dataset (shared)
$\left\{X_{P}^{\left[j\right]}\right\}$	The $j^{th}$ sample in $D_{p}$
$D_{d}^{r}$	$D_{p}$ permuted using the seed $\beta^{r}$
$D_{Aug}^{r}$	augmented public dataset. $D_{p}$ and $D_{d}^{r}$ weighted by $\alpha^{r}$.
$P_{i}^{r}$	is the accuracy of $f_{i}$ on $D_{t}$ at global round *r*
$S_{i}^{r}$	soft labels of $i^{th}$ client on $D_{Aug}^{r}$ at global round *r*
$S^{r}$	soft labels aggregated by the server at global round *r*
